# Traffic State Lane-Level Estimation Based on Transformer and Vehicle Trajectory Data

**DOI:** 10.3390/s26113376

**Published:** 2026-05-26

**Authors:** Wei Bai, Yan Zhao, Yanni Ju, Jing Gan, Linheng Li

**Affiliations:** 1 Department of Road Traffic Management, Sichuan Police College, Luzhou 646000, China; baiwei2019@scpolicec.edu.cn; 2 Intelligent Policing Key Laboratory of Sichuan Province, Sichuan Police College, Luzhou 646000, China; 3 School of Transportation, Southeast University, Nanjing 211189, China; 4 School of Modern Posts, Nanjing University of Posts and Telecommunications, Nanjing 210003, China

**Keywords:** traffic state lane-level estimation, vehicle trajectory data, transformer

## Abstract

Investigating the fundamental link between microscopic vehicular motion parameters and macroscopic traffic flow states is pivotal for advancing refined traffic state estimation research and propelling the progression of Intelligent Transportation Systems. In this paper, a basic Transformer model has been optimized and extended by incorporating embedding and pooling layers, and the model’s hyperparameters have been finely tuned through random search cross-validation. The creation of the Generalized Optimized Transformer (GOT) model ensued, where the multi-head attention mechanism adeptly encapsulates all spatiotemporal dynamics inherent in traffic data. Various benchmark models such as LSTM, RNN, and Transformer were put to test, each demonstrating unique performances in managing different traffic flow states. Among them, the GOT model exhibited superior performance, adeptly handling lane-level traffic state estimation tasks derived from microscopic vehicle trajectory data. In conclusion, this research elucidates the intricate and mutable mapping relationship between microscopic vehicular motion parameters and traffic flow states, proficiently executing lane-level traffic state estimation grounded on microscopic trajectory data. This paper is anticipated to provide fresh insights into the understanding of the complex relationship between microscopic vehicular motion parameters and traffic flow states.

## 1. Introduction

In modern traffic system management, accurate traffic state estimation is essential for understanding traffic operation, supporting refined traffic control, and improving the efficiency of Intelligent Transportation Systems. Traditional traffic state estimation methods usually rely on fixed detectors or aggregated traffic variables, such as traffic volume, average travel velocity, and occupancy [1,2]. Although these data sources are useful for describing section-level traffic conditions, they may be insufficient for capturing lane-level traffic variations and the microscopic behavioral mechanisms underlying traffic state transitions.

With the development of Vehicle-to-Everything (V2X), connected and automated vehicles, probe vehicles, and high-resolution trajectory sensing technologies, microscopic vehicle trajectory data have become increasingly available for traffic state estimation. Different from fixed detectors, vehicle trajectory data provide continuous vehicle-level motion information, including position, velocity, acceleration, and temporal evolution. These microscopic observations offer a new opportunity to infer macroscopic traffic flow states from individual vehicle behaviors. From the perspective of artificial intelligence and complex system modeling, macroscopic traffic states can be regarded as emerging from microscopic vehicle motion patterns. This micro-to-macro relationship is closely related to the concept of “supervenience”, which indicates that macroscopic properties depend on microscopic properties [3,4,5,6,7,8,9]. Therefore, an important question arises: can sparse microscopic vehicle trajectory observations be used to reliably identify lane-level traffic flow states?

Recent studies have explored the use of microscopic vehicle data for traffic state estimation and traffic parameter extraction. For example, intelligent connected vehicle data have been fused with fixed detector and probe vehicle data to estimate freeway lane-level traffic states [10]. Vehicle trajectory-based methods have also been used to reconstruct or impute traffic state information under sparse sensing conditions [11]. In addition, trajectory-driven network representation and deep learning models have been introduced to support traffic state prediction by extracting structural and temporal patterns from vehicle trajectory data [12]. Vehicle trajectory data have also been used for traffic parameter extraction and signal control optimization, further demonstrating their potential in traffic monitoring and management applications [13]. These studies indicate that microscopic vehicle trajectories can provide rich information for traffic state estimation.

However, several limitations remain in the existing literature. First, many traffic state estimation studies still focus on section-level, intersection-level, or network-level traffic conditions, while lane-level traffic state estimation based on sparse microscopic trajectory observations remains insufficiently explored. Second, although vehicle trajectories provide detailed motion information, the relationship between microscopic vehicle motion characteristics and macroscopic traffic state categories has not been sufficiently analyzed. Third, the availability and sparsity of trajectory observations can significantly influence traffic state estimation performance, especially when only partial connected vehicle or probe vehicle data are available [14]. Fourth, conventional machine-learning models and recurrent neural networks may have limited capability in capturing nonlinear dependencies, long-range temporal correlations, and salient local fluctuations in vehicle trajectory data.

To address these limitations, the core scientific question of this study is defined as follows: how can lane-level macroscopic traffic flow states be inferred from sparse microscopic vehicle trajectory observations by learning the nonlinear micro-to-macro relationship between individual vehicle motion patterns and aggregate traffic conditions? To answer this question, this study uses the processed vehicle trajectory data from the CitySim dataset to construct lane-level traffic state samples. Aggregate statistical analysis and autocorrelation analysis are first conducted to examine the distributional characteristics, volatility, stability, and temporal dependence of microscopic vehicle motion parameters under different traffic states. These analyses provide empirical evidence for the relationship between microscopic trajectory features and macroscopic traffic flow states.

To further learn this nonlinear relationship, this paper proposes a Generalized Optimized Transformer (GOT) model for lane-level traffic state estimation. Deep learning models provide a flexible way to capture complex mappings between microscopic trajectory features and macroscopic traffic states. CNNs can extract local features, but their ability to capture global temporal dependencies is limited [15]. RNNs and their variants, such as LSTM and GRU, can model sequential data, but they may suffer from gradient vanishing, gradient explosion, and limited long-range dependency modeling ability [16,17,18]. In contrast, the Transformer architecture uses the self-attention mechanism to capture global dependencies in sequential data and has shown strong representation capability [19]. Based on this advantage, the proposed GOT model extends the basic Transformer by enhancing feature representation, introducing an extended feed-forward network, adding a MaxPooling layer for salient local feature aggregation, and using randomized search for hyperparameter optimization.

The main contributions of this study are summarized as follows. First, this paper constructs a trajectory-based lane-level traffic state estimation framework that links microscopic vehicle motion parameters with macroscopic traffic flow states. Second, statistical and autocorrelation analyses are conducted to reveal the distributional and temporal characteristics of microscopic vehicle motion parameters under different traffic states. Third, a GOT model is proposed to learn the nonlinear micro-to-macro mapping from sparse microscopic trajectory observations to lane-level traffic state categories, including Free, Saturated, and Congestion states. Fourth, the proposed model is evaluated against benchmark models, including LSTM, RNN, and the basic Transformer, to verify its effectiveness in trajectory-based lane-level traffic state estimation.

The rest of the paper is organized as follows: Section 2 presents CitySim data processing and analysis of vehicle behavior under different traffic states. Section 3 introduces the Transformer and GOT models. Numerical experiments and analysis of the results are conducted in Section 4. Section 5 discusses the conclusion and potential future works.

## 2. Data Processing and Analysis

This research leverages a segment of the highway basic section derived from the CitySim dataset, which was unveiled in 2022. It should be noted that this study uses the processed vehicle trajectory data provided by the CitySim dataset rather than directly extracting trajectories from raw drone videos. Therefore, the effects of drone camera resolution, image quality, and object detection errors on trajectory extraction are not directly investigated in this study. In practical drone-based traffic monitoring, camera resolution may affect the accuracy of vehicle detection, localization, and trajectory reconstruction, especially for small or distant vehicles. The specific period analyzed spans from 17:20 to 18:03 on 22 May 2006, amounting to a total of 2024 s. This dataset encompasses approximately 6.38 million vehicle trajectory points, yielding an average of 1140 trajectory points per vehicle. Within various periods, the traffic state manifests certain fluctuations, with the maximum duration of vehicles’ presence within the drone video coverage area not exceeding 95 s.

### 2.1. Aggregate Statistical Analysis

To ascertain the supervenience relationship between microscopic vehicular motion parameters and macroscopic traffic states, traffic flow state parameters are extracted from the vehicle trajectory data provided by the CitySim dataset. The data are segmented into 14 batches based on the direction of road travel and time steps set at 5 min intervals. The 5 min aggregation interval was selected by considering both temporal resolution and statistical stability. Compared with longer intervals such as 10 min, a 5 min interval can better preserve short-term fluctuations and transitions among different traffic states. Compared with shorter intervals such as 2 min, it provides a sufficient number of vehicle observations within each aggregation window, making the estimated traffic volume, average velocity, and headway more statistically stable. Considering that the analyzed trajectory segment lasts approximately 2024 s, using a 5 min interval also allows the dataset to be divided into multiple comparable time windows while maintaining enough samples in each window for traffic state classification. Therefore, the 5 min interval provides a practical balance between capturing traffic state dynamics and reducing random fluctuations caused by insufficient samples.

Table 1 presents an elaborate exposition of the statistical characteristics of vehicle flow, average driving velocity, and headway for each batch.

Freeway i,j, where a contains the numbers 1–7, i representing different road locations, and j contains 1 and 2, with 1 representing vehicles travelling up and 2 representing vehicles travelling down.

In this research, the K-means clustering method was employed for unsupervised traffic state segmentation based on the aggregated traffic flow indicators, including traffic volume, average travel velocity, and headway. The traffic status categories in Table 2 were not manually assigned according to the included data sets. Instead, they were obtained from the clustering results and then interpreted according to their traffic flow characteristics.

Specifically, the cluster characterized by relatively high travel velocity and large headway was labeled as Free, indicating that vehicles can travel with relatively weak interactions. The cluster with intermediate traffic characteristics was labeled as Saturated, representing a transitional state in which traffic demand increases and vehicle interactions become stronger. The cluster with lower travel velocity, smaller headway, and higher traffic concentration was labeled as Congestion.

It should be noted that the present study did not define Free, Saturated, and Congestion states using predefined threshold ranges. Instead, the categories were derived from K-means clustering results. Therefore, the values reported in Table 2 should be interpreted as representative cluster characteristics rather than fixed universal classification thresholds. The numerical characteristics of these categories are data-dependent and may vary across different road sections, observation periods, or datasets.

**Figure 1:**
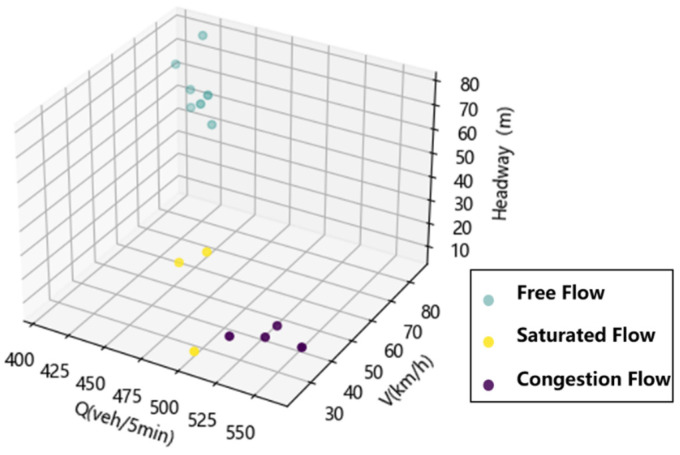
Traffic state classification of the examined section.

The results of the traffic state segmentation are summarized in Table 2 and visualized in Figure 1. Table 2 reports the representative statistical characteristics of each clustered traffic state, while Figure 1 provides an intuitive visualization of the clustering results in the traffic-flow feature space. As shown in Figure 1, the three traffic states present distinguishable distributional patterns, supporting the use of Free, Saturated, and Congestion as data-driven labels for the subsequent traffic state classification task.

#### 2.1.1. Concentrated Trend Analysis

Concentrated trend analysis, alternatively known as central tendency analysis, is a prevalent statistical technique utilized to characterize data set distributions. The primary goal of this segment is to evaluate the centrality and span of “typical values” within a data set through the application of centralized trend analysis. Metrics chosen for this portion encompass mean, median, skewness, kurtosis, and Jarque–Bera test values.

(1) $$Skewness=E\left[{\left((x-\mu )/\sigma \right)}^{3}\right]$$

(2) $$Kurtosis=E\left[{\left(\left(x-\mu \right)/\sigma \right)}^{4}\right]-3$$

(3) $$Jarque-Bera=\frac{n}{6}\left(Skewness^{2}+{\left(Kurtosis-3\right)}^{2}/4\right)$$

where E denotes the expectation operation, x denotes the sample of data, μ denotes the mean of the sample, σ denotes the standard deviation of the sample, and n denotes the number of samples.

#### 2.1.2. Discrete Trends Analysis

Discrete trends analysis, also known as variability analysis, is a technique employed to gauge the width, breadth, and degree of dispersion in a data distribution. The width of a data distribution represents the distance between the maximum and minimum values of the data. The breadth of a data distribution pertains to the range of data distribution, typically measured by the interquartile range. The dispersion of a data distribution is the average deviation of data values relative to the mean. The measures of dispersion selected for this section encompass the range, interquartile range, standard deviation (SD), mean absolute deviation (MAD), and coefficient of variation (CoV). The corresponding formulas are shown in Equations (4)–(6), and the meanings of the variables involved in these formulas are consistent with those defined in Equations (1) and (2).

(4) $$SD=\sqrt{\sum (x-\mu {)}^{2}/n}$$

(5) $$MAD=\frac{\sum |x-\mu |}{n}$$

(6) $$CoV=\frac{SD}{MAD}\times 100\%$$

**Figure 2:**
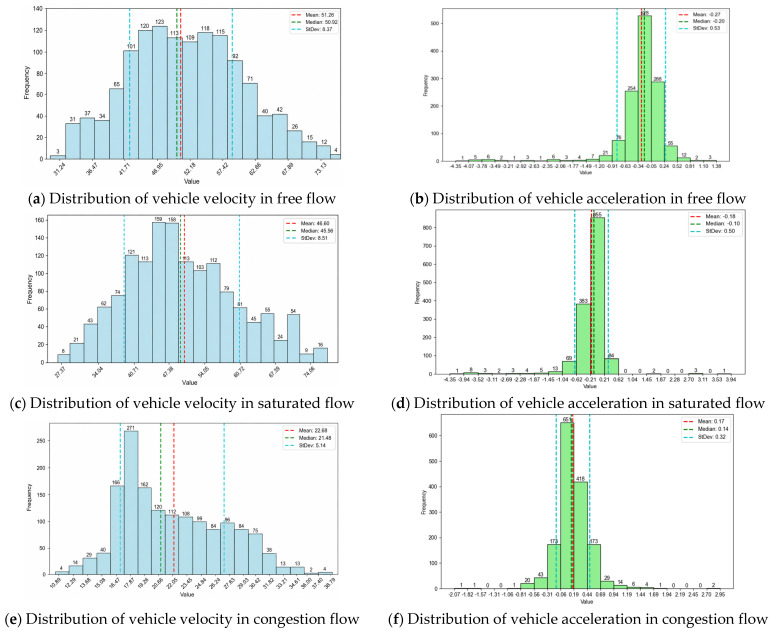
Distribution of microscopic vehicle motion parameters.

The distribution of individual vehicle average velocity data and average acceleration data under various traffic conditions is depicted in Figure 2, with specific values detailed in Table 3.

In the centralized trend analysis, the velocity data during the free flow state primarily cluster in the high value zone, displaying an approximately symmetrical distribution. Conversely, in the congested flow state, velocities predominantly aggregate in the low value zone, demonstrating considerable asymmetry. Vehicle velocities across all traffic states exhibit right skewness, particularly pronounced in the congested flow state, where both the right skewness and kurtosis reach peak values. The Jarque–Bera test indicates that none of the data conform to a normal distribution, with data in the congested flow state demonstrating the highest degree of deviation from normal distribution. This non-normality suggests that microscopic vehicle motion parameters exhibit skewed, heavy-tailed, and state-dependent distributional characteristics. Therefore, a simple linear mapping or a model relying on Gaussian-like assumptions may be insufficient to capture the complex relationship between microscopic vehicle behavior and macroscopic traffic states. This non-normality suggests that microscopic vehicle motion parameters exhibit skewed, heavy-tailed, and state-dependent distributional characteristics. Therefore, a simple linear mapping or a model relying on Gaussian-like assumptions may be insufficient to capture the complex relationship between microscopic vehicle behavior and macroscopic traffic states. It should be noted that this distributional analysis does not by itself demonstrate the superiority of a specific deep learning architecture. Rather, it provides empirical evidence that the traffic state estimation task may benefit from flexible nonlinear modeling methods that can represent complex feature interactions in microscopic trajectory data.

Discrete trend analyses revealed that the range of variation in vehicle velocities was wider for the free flow and saturated flow states, while the range of variation was narrowest for the blocked flow state. The most considerable degree of data dispersion was observed in the free flow, while the least dispersion was seen in the congested flow. The highest coefficient of variation was discovered in the congested flow, signifying that its data were the most dispersed relative to the mean value.

In short, for the free flow condition, velocity data gravitate towards higher value ranges, presenting rightward skewness and elevated dispersion characteristics. This could be attributed to variations in driving styles and the imposition of an upper velocity limit. Concurrently, the high dispersion of acceleration in free-flow conditions underscores the driver’s liberty to adjust velocity. In the saturated flow state, the velocity data display high symmetry and dispersion, potentially due to traffic flow fluctuation. This oscillation necessitates drivers to frequently modify their velocity, thereby expanding the velocity variation range. Similarly, acceleration under saturated flow conditions exhibits high dispersion and frequent alterations. In the congestion flow condition, velocitys primarily gather in the lower value range, exhibiting less dispersion, yet substantial vehicular travel disparities exist. Additionally, acceleration in congested flow shows limited dispersion and concentrated data, mirroring the attempts of vehicles to accelerate to traverse the blockage as swiftly as possible.

### 2.2. Autocorrelation Analysis

The Hurst index is an index that describes the autocorrelation of variables and the strength of dependencies [20]. This index is employed to assess the autocorrelation of vehicle velocity and acceleration. This research utilizes rescaled range analysis (R/S analysis) for linear fitting analysis to accomplish the calculation of the Hurst index [21]:

(7) $$H=\frac{\ln(N)}{\ln(2)}-\frac{\ln(R/S)}{\ln(2)}$$

where H is the obtained Hurst index; N is the total number of data sets; R is the range of data set values, i.e., the difference between the maximum data value and the minimum data value; S is the data set standard deviation.

This research employed the Hurst Index for autocorrelation assessment following these steps:

(1) Decompose the data into multiple subsequences using a sliding window.

(2) Calculating the mean and sum of squared deviations for each subsequence.

(3) Perform a linear fit with the sum of squared deviations serving as the dependent variable and the length of the subsequence as the independent variable.

(4) Computing the slope of the linear fit on each scale, which equates to the Hurst exponent.

**Figure 3:**
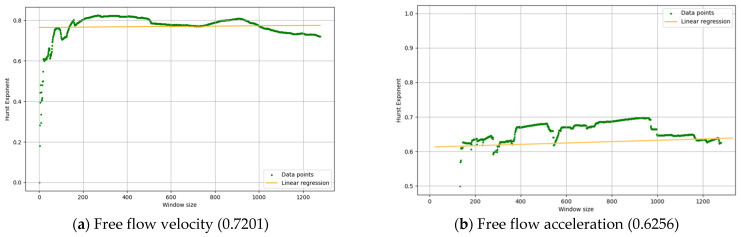

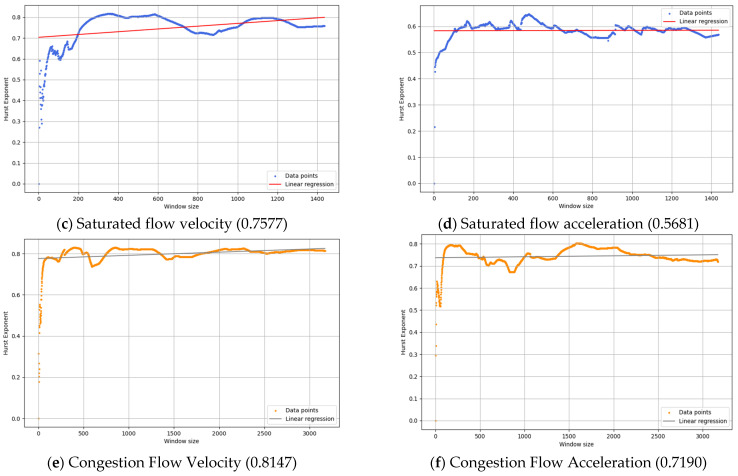
Linear fit of Hurst exponent.

The Hurst exponent varies between 0 and 1. Values leaning towards 1 denote a strong positive correlation; values tending towards 0 signal a significant negative correlation. Values near 0.5 suggest the absence of autocorrelation in the data set, that is, the data is distributed randomly. Figure 3 provides an in-depth illustration of the Hurst exponent’s fluctuation in the velocity and acceleration data series under diverse traffic conditions.

The Hurst exponent for both velocity and acceleration data surpasses 0.5 under free flow, saturated flow, and congested flow conditions, signifying substantial autocorrelation. This implies that both velocity and acceleration data display a positive correlation at the selected scales. In the saturated flow state, the Hurst exponent for acceleration slightly exceeds 0.5 (0.5681), indicating negligible variation in acceleration. On the whole, vehicles may deviate from normal driving norms at a certain point in time or space, leading to individual vehicle behavior deviating from the macroscopic traffic state, but it remains non-random.

These findings offer preliminary data support for traffic flow state estimation based on microscopic vehicle motion parameters, emphasizing the intimate link between microscopic vehicle motion parameters and macroscopic traffic states. Simultaneously, it uncovers the variability of vehicle behaviors under identical traffic states, suggesting that the accuracy of traffic state estimation based on microscopic vehicle motion parameters alone via statistical analysis falls short of meeting everyday traffic system management needs.

The above feature analysis provides an empirical basis for the subsequent modeling considerations. First, the statistical differences in velocity and acceleration under different traffic states indicate that microscopic vehicle motion parameters contain discriminative information for traffic state classification. Second, the non-normal and state-dependent distributions suggest that simple linear or Gaussian-assumption-based models may be insufficient to describe the micro-to-macro mapping relationship. Third, the Hurst index results show that vehicle velocity and acceleration exhibit temporal dependence rather than purely random fluctuations, indicating that sequential modeling is necessary. These findings support the use of nonlinear sequence modeling in general. However, they do not uniquely validate the proposed GOT architecture. The specific effectiveness of the GOT components is therefore further examined through benchmark comparison, ablation study, and pooling sensitivity analysis in Section 4.

## 3. Methods

Based on the feature analysis in Section 2, the model construction in this study follows three considerations. First, the significant differences in microscopic motion parameters among Free, Saturated, and Congestion states indicate that vehicle trajectory features can provide useful information for lane-level traffic state estimation. Second, the non-normal and nonlinear distributional characteristics of velocity and acceleration require a flexible learning model rather than a simple linear classifier. Third, the autocorrelation results suggest that vehicle motion parameters have temporal dependence, which should be explicitly modeled.

Based on the feature analysis in Section 2, the model construction in this study follows three modeling considerations. First, the significant differences in microscopic motion parameters among Free, Saturated, and Congestion states indicate that vehicle trajectory features can provide useful information for lane-level traffic state estimation. Second, the non-normal and nonlinear distributional characteristics of velocity and acceleration suggest the need for flexible nonlinear representation. Third, the autocorrelation results suggest that vehicle motion parameters have temporal dependence, which should be explicitly modeled. These findings motivate the use of nonlinear sequence modeling. Among possible model choices, this study adopts a Transformer-based framework because its self-attention mechanism is suitable for learning temporal dependencies in trajectory sequences. The task-specific modifications of GOT are then evaluated empirically in Section 4 rather than being assumed solely from the statistical analysis.

### 3.1. Problem Description

**Figure 4:**
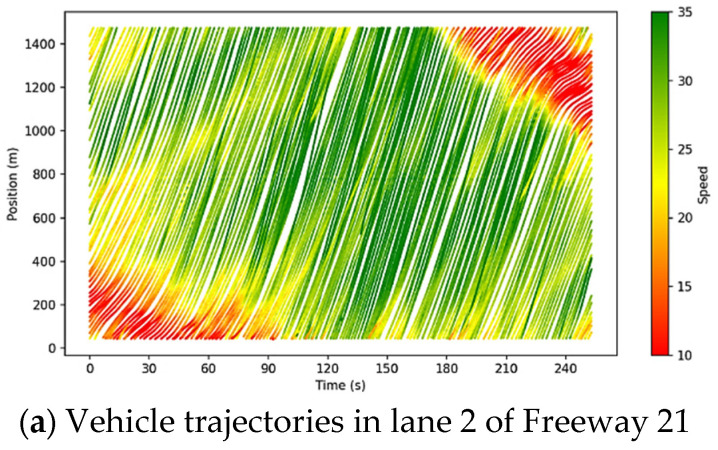

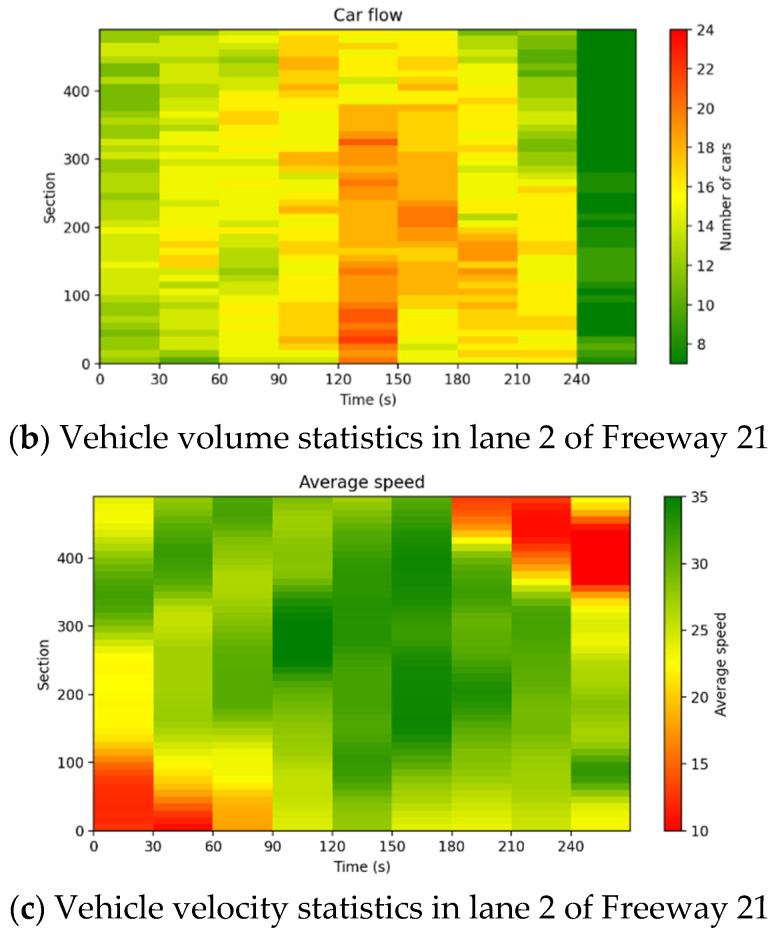
Example of trajectory-based lane-level traffic flow extraction in lane 2 of Freeway 21.

This research explores the application of microscopic vehicular motion data, represented in the form of vehicle trajectories. Given that CitySim contains comprehensive trajectory data of all vehicles traversing the corresponding road segment, this research utilizes CitySim to generate simulated fixed detector flow and velocity data. Taking the vehicles in lane 2 of Freeway Section 2 as an example, Figure 4 illustrates how microscopic vehicle trajectory data are transformed into lane-level traffic flow observations. It should be noted that Figure 4 is not a spectrogram. Instead, it presents the time-space distribution of vehicle trajectories and the corresponding traffic parameters obtained from simulated fixed detectors. Specifically, Figure 4a shows the vehicle trajectory diagram in the time-space plane, where each curve represents the movement trajectory of an individual vehicle along the lane. The horizontal axis denotes time, and the vertical axis denotes the longitudinal position along the road section. The slope of each trajectory reflects the vehicle velocity. Denser trajectories and smaller spacing between curves indicate higher traffic concentration. Figure 4b,c show the lane-level traffic volume and average velocity obtained by aggregating the trajectory data within fixed temporal and spatial intervals. These two subfigures are used to demonstrate how microscopic trajectory records can be converted into macroscopic lane-level traffic flow indicators. Therefore, Figure 4 provides an intuitive example of the connection between vehicle trajectory data and traffic flow state estimation.

**Figure 5:**
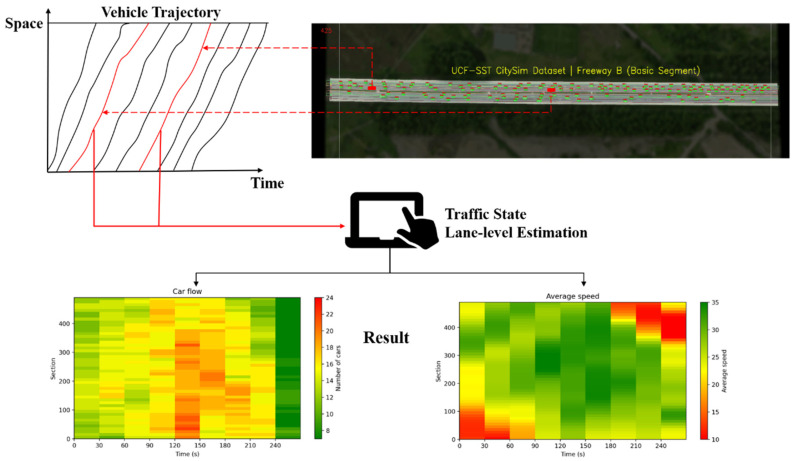
Traffic state estimation process driven by vehicle trajectory data.

In this research, vehicle trajectory data is extracted randomly at a certain proportion, followed by the usage of micro-vehicle data to accomplish traffic state estimation. The specific implementation is demonstrated in Figure 5.

### 3.2. Basic Transformer

**Figure 6:**
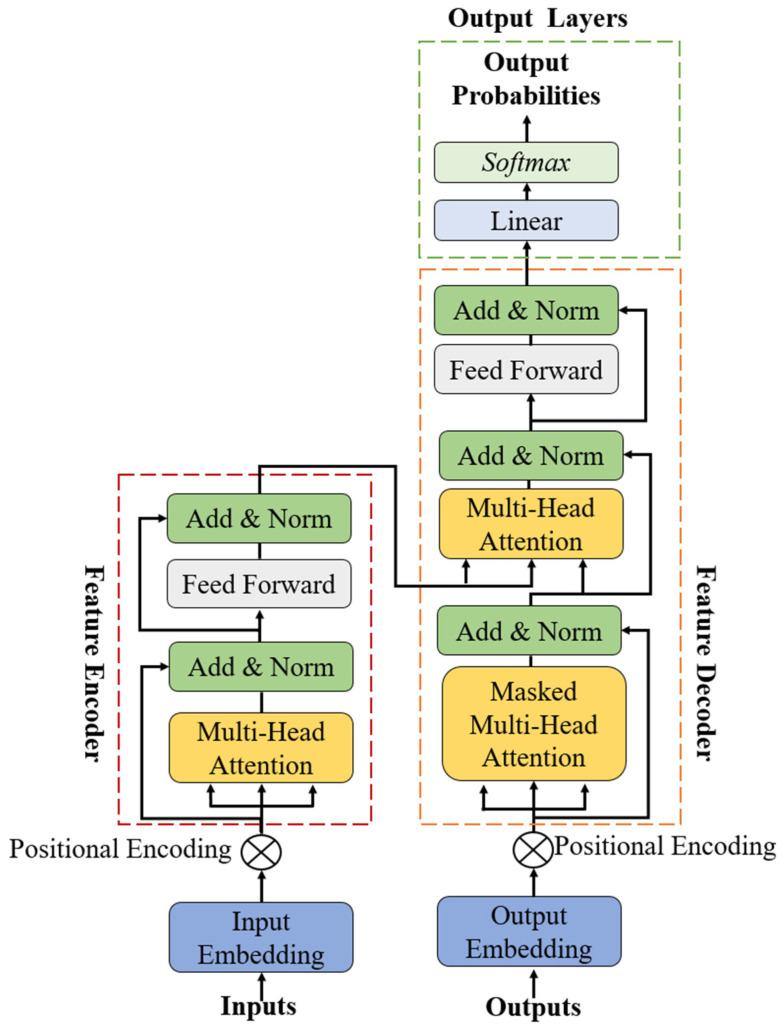
Structure of the base Transformer model.

The Transformer model hinges on the self-attention mechanism for its encoding and decoding processes [22]. Its basic architecture is made up of four parts: input layer, feature encoder, feature decoder, and output layer [23]. This is depicted in Figure 6.

#### 3.2.1. Input Layer

The Transformer model initially carries out input and output embeddings. The research presented here pertains to a multi-step multivariate time series problem. The model needs to leverage multiple input sequences (trajectory data of several vehicles) to estimate several output sequences (traffic state parameters of numerous road sections). The input consists of the trajectory data, which includes time points, position data, and velocity data of several vehicles. The output of the model is the lane-level traffic state category, including Free, Saturated, and Congestion. Macroscopic traffic flow parameters, such as traffic volume, average travel velocity, and headway, are used to construct the corresponding traffic state labels rather than being directly predicted as continuous outputs. Both the input and output data in this problem present high dimensionality and intricate structure. As these are continuous values, operations akin to word embedding are unnecessary. However, other processing steps are required to adapt the model to the data’s format and dimensions.

We divide the trajectory data into multiple spatiotemporal tensor samples according to fixed time windows. In this study, each time window lasts 30 s. For each lane and each time window, microscopic trajectory observations are organized into an input tensor to represent the local traffic evolution within the lane.

To avoid ambiguity, it should be noted that the term “batch size” in model training is different from the number of vehicles included in each input sample. The training batch size is a hyperparameter that determines how many input samples are used in one parameter update. In contrast, the number of vehicles in each input sample refers to the sampled microscopic trajectory observations used to estimate the lane-level traffic state. In this study, two vehicles are randomly selected within each lane-time window to construct one input sample. This setting is used as a baseline sparse-observation scenario to simulate the situation where only a limited number of connected or probe vehicles are available for lane-level traffic state estimation. The influence of the number of sampled vehicles can be further examined in future studies by varying the probe vehicle penetration rate.

The input tensor is represented as (B, T, S, F), where B denotes the training batch size, T denotes the temporal sequence length, S denotes the number of discretized spatial segments in the lane, and F denotes the feature dimension. Specifically, T is set to 300 time steps, corresponding to a 30 s observation window. The lane section is discretized at an interval of 30 m to form the spatial dimension S. The feature dimension F contains microscopic vehicle motion features, such as vehicle velocity. For each input sample, the trajectory features of the sampled vehicles are mapped into the corresponding time-space cells according to their timestamps and longitudinal positions. If multiple vehicle observations fall into the same cell, their feature values are aggregated. If no observation is available in a cell, a padding and masking strategy is applied to prevent the model from treating missing values as valid observations.

The lane-level traffic state label is obtained from the aggregated traffic flow parameters of the corresponding lane-time window, including average velocity, traffic volume, and headway. Therefore, the input tensor represents sparse microscopic trajectory observations, while the output label represents the macroscopic lane-level traffic state of the same lane-time window, including Free, Saturated, and Congestion states.

Through this representation, sparse microscopic vehicle trajectory observations can be converted into a structured time-space tensor, which serves as the model input for estimating lane-level traffic states, including Free, Saturated, and Congestion states.

Subsequently, the output 2D tensor is constructed with the shape (section size, output dim): section size is the location of the road section, and output dim encapsulates features including velocity and flow rate. Considering that different vehicles first appear at varying times, a padding strategy is adopted to address this: special values are added after the shorter sequences to match the length of the longest sequence. To circumvent the potential risk of the model treating padding values as valid information, we employ a masking technique to disregard the padding values.

The Transformer model lacks any inherent recurrent or convolutional structure; it requires additional positional information to comprehend the sequence order. In the context of time-series data, positional encoding can be employed to denote the position of a data point within the series, i.e., a point in time. The model takes the input—vehicle trajectory data—and encodes the time points as positions, thereby understanding the order of the data points within the time series, i.e., the temporal sequence of vehicle trajectories. Similarly, it discerns the order of output data within the time series, i.e., the temporal sequence of the macroscopic traffic flow. The computational process for positional encoding is represented in Equation (8) [24]:

(8) $$\begin{array}{l} PE_{\left(p,2i\right)}=\sin\left(\frac{p}{10000^{2i/dmodel}}\right)\\ PE_{\left(p,2i+1\right)}=\cos\left(\frac{p}{10000^{2i/dmodel}}\right) \end{array}$$

where p denotes the time point of the input data, i denotes the corresponding feature dimension, each input data is computed using sin and cos functions with different periods, and the wavelength is gradually increased from 2π to 10000×2π.

#### 3.2.2. Feature Encoder and Feature Decoder

The feature encoder is in charge of applying feature encoding to the vehicle motion parameters following positional encoding, and then passing these encoded features to the feature decoder. The feature decoder takes the output from the feature encoder, decodes it, and sends it to the output layer. The encoder or decoder may contain multiple identical encoder or decoder layers, each equipped with a multi-head self-attention module, a feed-forward neural network module, and several normalization and residual connection modules.

The multi-head self-attention mechanism is the core of the Transformer model. Given that this research involves micro-to-macro mapping, the self-attention mechanism facilitates the model in learning long-range dependencies in the time-series data. This understanding helps the model to recognize how the time points in vehicle trajectory data are interconnected and collectively influence the traffic state parameters of a road segment. Through the self-attention mechanism, the model can learn the mapping relationship from the vehicle trajectory data to the traffic state parameters of a road segment, enhancing the model’s ability to accurately estimate traffic state parameters.

Following each step of the self-attention and feed-forward neural network processes, the Transformer model carries out layer normalization and residual connections. This aids in stabilizing the model’s output and accelerating the training process. The output from the self-attention mechanism is routed into a feed-forward neural network, which is independently applied at each position. This feedforward network is a two-layer fully connected network, where the intermediary layer is activated via a Relu function.

(9) $$FFN(x)=\max(0,xB_{1}+b_{1})B_{2}+b_{2}$$

where  x is the normalized output matrix of the attention layer, B is the weight vector, b is the bias term, and max(0,·) is the Relu activation function.

#### 3.2.3. Output Layer

The output layer processes the decoder features through a fully connected layer and a Softmax layer, producing the predicted probabilities of the three traffic state categories: Free, Saturated, and Congestion.

### 3.3. Generalized Optimized Transformer (GOT)

**Figure 7:**
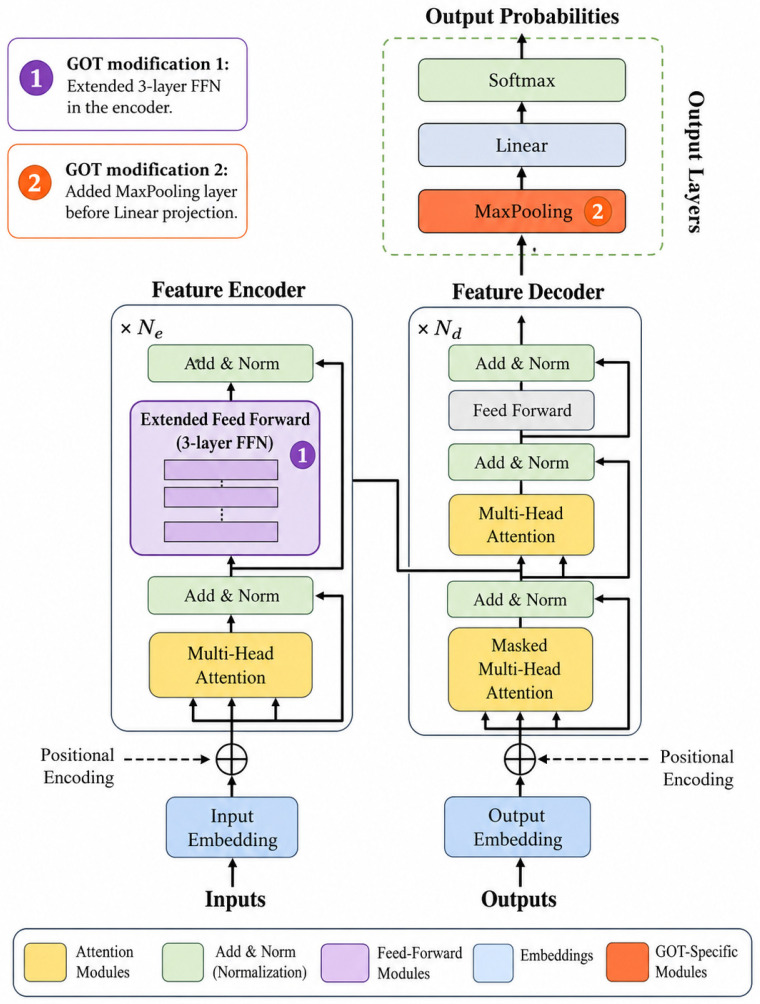
Structure of the Generalized Optimized Transformer (GOT) model.

In an effort to extend the Transformer model beyond its traditional use in Natural Language Processing (NLP) tasks, and to accommodate the specific requirements of lane-level traffic flow state estimation based on microscopic vehicle motion parameters, we have refined and optimised the Transformer. The result is the Generalized Optimized Transformer (GOT) model, which is depicted in Figure 7.

The feature analysis in Section 2 indicates that microscopic vehicle velocity and acceleration show distinguishable, non-normal, state-dependent, and temporally correlated patterns under different traffic states. These findings suggest that lane-level traffic state estimation requires a model capable of representing nonlinear feature interactions and temporal dependencies. However, these statistical findings should be interpreted as general modeling motivation rather than direct proof of Transformer superiority. Accordingly, the GOT model is proposed as one possible nonlinear sequence modeling framework, and its effectiveness is evaluated through benchmark comparison, ablation analysis, and pooling strategy sensitivity analysis in Section 4. In this framework, the self-attention module is used to model temporal dependencies in trajectory sequences, the extended FFN improves nonlinear feature transformation, and the MaxPooling layer preserves prominent local responses that may be associated with traffic state transitions.

#### 3.3.1. Extension of Feed Forward Network (FFN)

This study enhances the Feed Forward Network (FFN) component of the fundamental Transformer model. Our augmented model expands the feed-forward neural network hierarchy to three layers by introducing an extra fully connected layer (Dense Layer) between the existing two fully connected layers. This extra layer enriches the model’s depth, enabling it to learn more complex features and patterns. Consequently, the feed-forward neural network for our enhanced model is given by Equation (10):

(10) $$FFN(x)=\max(0,\max(0,xB_{1}+b_{1})B_{2}+b_{2})B_{3}+b_{3}$$

where $B_{3},b_{3}$ are the weights and bias terms of the additional fully connected layers.

The additional FFN layer is introduced to enhance the nonlinear representation capability of the model. In the present lane-level traffic state estimation task, the mapping from microscopic vehicle trajectory features to macroscopic traffic states is highly nonlinear. Local variations in vehicle velocity and acceleration may correspond to different traffic states depending on the surrounding traffic conditions. Therefore, the third FFN layer is expected to improve the model’s ability to capture complex micro-to-macro mapping relationships after the self-attention operation. To verify its specific contribution, an ablation study is further conducted in Section 4.3.

#### 3.3.2. MaxPooling Layer

Microscopic vehicle trajectory data usually contain local and short-term fluctuations caused by heterogeneous driving behaviors, such as abrupt velocity reductions, acceleration changes, and stop-and-go movements. These local variations may provide important cues for distinguishing different lane-level traffic states. After the Transformer module, a MaxPooling layer is introduced to aggregate salient local feature responses and reduce redundant feature information. Compared with Average Pooling, which tends to smooth local feature variations, MaxPooling preserves the most prominent response within each local feature region. This property is useful for retaining critical motion-related features that may correspond to traffic state transitions. In addition, the pooling operation reduces the feature dimension, thereby decreasing computational complexity and memory consumption. It should be noted that MaxPooling is not assumed to be theoretically optimal for all trajectory-based traffic state estimation tasks. Instead, it is adopted in this study as a simple and effective local feature aggregation strategy. To further evaluate its contribution, an ablation and sensitivity analysis is conducted in Section 4.3, where MaxPooling is compared with no pooling, Average Pooling, and Attention-based Pooling. In the MaxPooling layer, the model performs a pooling operation within each local feature region and outputs the maximum value in that region, which can be expressed as:

(11) $$y_{ij}^{k}=\underset{(p,q)\in R_{ij}}{\max}x_{ij}^{k}$$

where $y_{ij}^{k}$ represents the maximum pooled output value associated with the *k*-nd feature in rectangular region $R_{ij}$, and $x_{ij}^{k}$ represents the sample located at (p,q) in the rectangular region $R_{ij}$.

#### 3.3.3. Overfitting Prevention

Preventing overfitting is a crucial aspect in the training of neural network models. In our model, we ensure that the model has good generalisation performance on data other than the training set by introducing L2 regularisation and early stopping strategies. L2 regularisation is a method to prevent overfitting by adding a weight sum-of-squares term to the loss function. Specifically, for a network of weight parameters B, the loss function L with L2 regularisation can be expressed as Equation (12):

(12) $$L=L_{0}+\lambda \|B{\|}^{2}$$

where L0 is the original unregularised loss, α is the L2 regularised strength parameter, and $\|B{\|}^{2}$ is the parameter of the weight parameter B (i.e., the sum of the squares of all elements of the weight matrix B). By introducing this additional penalty term, we can encourage the model to learn as few weights as possible, thus preventing the model from over-relying on particular features.

Meanwhile, we use Early Stopping and Model Checkpoint to prevent overfitting. Early Stopping calculates the performance of the model on the validation set at the end of each training cycle (epoch). If the model’s validation performance is not improved for several consecutive epochs, we terminate the training early. The model checkpoint then saves the model with the lowest validation loss after each epoch, ensuring that we always use the best-performing model. The procedure is shown in Algorithm 1.
**Algorithm 1.** Early Stopping Method Anti-Fitting AlgorithmSet the number of cycles in which validation performance is not improved to patience Initialise best val_loss to positive infinity Initialise patience counter to 0**BEGIN**
**1**Performed for each training epoch:**2**Training the model Calculate validation set loss val_loss**3**If val_loss < best val_loss:    Update best val_loss = val_loss    Save the current model state  Reset patience counter to 0**4**else:    patience counter += 1**5**If patience counter >= patience:    Early termination of the training**END**


Algorithm 1 describes that at the end of each training epoch, we calculate the model’s loss on the validation set, and if the validation set loss is not improved for “patience” consecutive cycles, then we terminate training early.

#### 3.3.4. Parameter Optimisation

When designing a deep learning model, choosing the appropriate hyperparameters has a critical impact on the model’s performance. We adopt Randomized Search for hyperparameter optimisation. Randomised Search differs from the classical Grid Search, which systematically explores every possible combination of parameters in the parameter space. Instead, Randomised Search evaluates a given number of parameter combinations by randomly selecting parameter values from a predefined parameter distribution. Stochastic search is more efficient compared to Grid Search in large parameter spaces and can find better hyperparameter combinations with limited computational resources and time.

Specifically, assuming that we have d hyperparameter with n possible values for each hyperparameter, the grid search needs to try $n^{d}$ parameter combinations. For stochastic search, we can preset a budget of $k\left(k<n^{\alpha }\right)$ and evaluate only k parameter combinations, greatly reducing the computational effort of the search. This strategy shows high efficiency when dealing with large-scale parameter spaces and can find better parameter combinations within limited computational resources and time.

To ensure a fair comparison among different models, the randomized search strategy was applied to all models in this study, including LSTM, RNN, the basic Transformer, and the proposed GOT model. For each model, the hyperparameters were selected from predefined candidate ranges, and the same search budget was adopted. The final hyperparameter settings used in the experiments correspond to the configuration that achieved the lowest validation loss on the validation set. Therefore, the performance comparison among the benchmark models and the proposed GOT model was conducted under the same hyperparameter optimization protocol.

## 4. Results

### 4.1. Benchmark Experimental

Since the lane-level traffic state estimation task is formulated as a three-class classification problem, including Free, Saturated, and Congestion states, categorical cross-entropy was consistently adopted as the loss function for all models, including LSTM, RNN, the basic Transformer, and the proposed GOT model. This setting ensures that all models are optimized under the same classification objective. Accuracy and validation loss were used to evaluate the overall training performance, while confusion matrix, precision, recall, and F1-score were further reported to assess class-wise classification performance.

For a fair comparison, all models were trained and evaluated using the same data partition, input features, validation strategy, and early stopping criterion. In addition, the hyperparameters of all benchmark models and the proposed GOT model were optimized using the same randomized search procedure described in Section 3.3.4. The candidate hyperparameters included the optimizer type, learning rate, batch size, number of hidden units, dropout rate, regularization coefficient, and model-specific structural parameters. For each model, the configuration with the lowest validation loss was selected as the final setting for test evaluation.

The LSTM model consists of an LSTM layer with 100 hidden units and an input shape of (95, 2), followed by two fully connected layers. The first layer has 64 cells and incorporates regularization. The second layer has 3 units and uses a softmax activation function. The Adam optimizer was used with a learning rate of 0.001. Since the lane-level traffic state estimation task is a three-class classification problem, categorical cross-entropy was used as the loss function. Early stopping is implemented, ceasing training if there is no improvement in the validation set loss across 3 consecutive epochs. Training runs for a maximum of 20 epochs with a batch size of 32.

The RNN model is constructed using Keras’ Sequential API. It incorporates two simple RNN layers, each with 128 units and uses regularization to prevent overfitting. Each RNN layer is followed by a dropout layer, with a dropout rate of 0.5. A fully connected output layer with 3 units and a Softmax activation function was used to output the probabilities of the three traffic states. Categorical cross-entropy was used as the loss function, and RMSProp was adopted as the optimizer with a learning rate of 0.001.

The Transformer model consists of multiple Transformer blocks and a global average pooling layer. The RMSprop optimizer is used with a learning rate set at 0.001. Categorical cross-entropy was used as the loss function, and accuracy was used as the overall performance metric. Training utilizes a batch size of 16 and runs for a maximum of 10 epochs.

The detailed model configurations and training parameters are summarized in Table 4. To improve reproducibility, the table reports the exact number of recurrent layers, Transformer blocks, attention heads, hidden units, dropout rates, optimizers, learning rates, training epochs, and batch sizes used for each model.

It should be noted that the optimizer type for each model was determined through the same randomized hyperparameter search procedure rather than manually fixed in advance. For the proposed GOT model, SGD was selected as the final optimizer because it achieved the lowest validation loss among the tested optimizer candidates under the same search budget. This setting was therefore adopted to ensure that the final configuration of each model corresponded to its best validation performance.

### 4.2. Iteration of Loss and Accuracy

**Figure 8:**
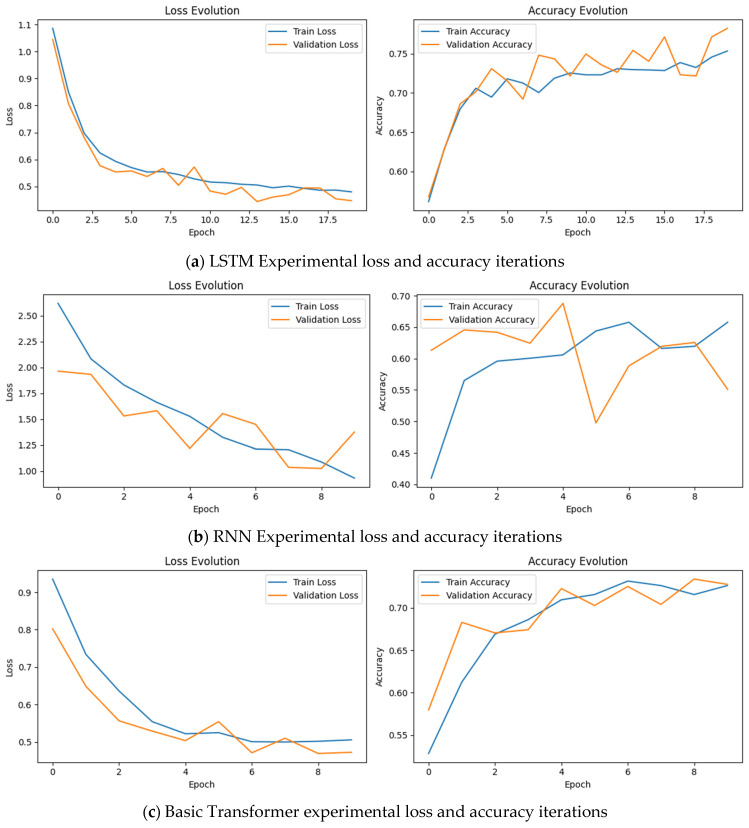

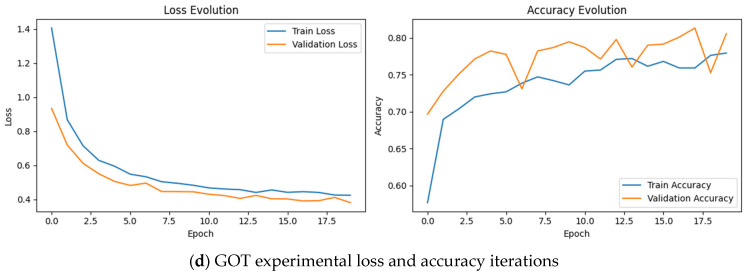
Comparison of accuracy and loss iteration.

In this paper, data from the comprehensive citysim dataset is leveraged to evaluate the performance of the various models. The learning process of each model is closely monitored by plotting iterations of accuracy and loss. This approach not only provides a clear visualization of each model’s learning process but also helps identify the optimal moment to halt training. For each of the four model variants, we elected to terminate the training phase when we observed the onset of decreasing accuracy in the training set. The iterations of loss and accuracy for each model on the test set are comparatively assessed and visually represented in Figure 8.

Figure 8 presents a comparison of the losses of the four model variants. Notably, the GOT model exhibits the smallest overall loss, falling below 0.4. Moreover, it is the only model that continues to decrease in loss, while the other three models show a slight uptick in loss. In terms of accuracy, the RNN model registers the lowest overall accuracy, while the GOT model secures the highest accuracy, standing at 80.23%. The accuracy of each model on the test set initially undergoes a significant decline before rebounding, with the RNN model manifesting the highest degree of fluctuation, the LSTM model displaying the highest frequency of fluctuations, and the Transformer model showcasing the least magnitude and frequency of fluctuations. However, the Transformer model’s accuracy is slightly lower than that of the GOT model. Judging both by the loss and accuracy metrics, the GOT model demonstrates a superior performance.

Although accuracy and validation loss provide an overall evaluation of model performance, they may not fully reflect the classification performance for each traffic state, especially when the data distribution among Free, Saturated, and Congestion states is imbalanced. Therefore, to provide a more comprehensive evaluation of the three-class traffic state estimation task, a confusion matrix and class-wise metrics, including precision, recall, and F1-score, are further introduced in Section 4.4.

### 4.3. Ablation and Sensitivity Analysis

To further examine the specific contribution of the proposed structural modifications, an ablation study was conducted for the GOT model. Four model variants were compared, including the Basic Transformer, Transformer with the third FFN layer, Transformer with MaxPooling, and the full GOT model. All variants were trained and tested under the same dataset partition, input features, and evaluation settings. The results are shown in Table 5.

The results show that the Basic Transformer achieves an accuracy of 76.85% with a validation loss of 0.462. After adding the third FFN layer, the accuracy increases to 78.12%, and the validation loss decreases to 0.438. This indicates that the additional FFN layer improves the nonlinear feature representation capability of the model. When only MaxPooling is introduced, the accuracy further increases to 78.64%, and the validation loss decreases to 0.421, suggesting that MaxPooling helps preserve salient local trajectory features related to traffic state transitions. The full GOT model achieves the highest accuracy of 80.23% and the lowest validation loss of 0.387, indicating that the third FFN layer and MaxPooling provide complementary benefits.

In addition, a sensitivity analysis was conducted to compare different pooling strategies. As shown in Table 6, the model without pooling achieved an accuracy of 78.12% and a validation loss of 0.438. Average Pooling improved the accuracy to 78.57% and reduced the validation loss to 0.426, indicating that pooling-based feature aggregation is beneficial. MaxPooling achieved the best performance, with an accuracy of 80.23% and a validation loss of 0.387. Attention-based Pooling also performed well, achieving an accuracy of 79.84% and a validation loss of 0.395. However, it introduced additional trainable parameters and higher model complexity. Therefore, MaxPooling was adopted in the proposed GOT model because it provides a favorable balance among estimation accuracy, feature saliency preservation, and model simplicity.

Overall, the ablation and sensitivity results provide empirical evidence that the proposed GOT structure improves model performance not only by increasing network depth but also by enhancing the aggregation of salient local trajectory features. These results support the effectiveness of the third FFN layer and the MaxPooling layer in the lane-level traffic state estimation task.

### 4.4. Confusion Matrix and Class-Wise Evaluation

To further evaluate the classification performance of the proposed GOT model for different traffic states, a normalized confusion matrix and class-wise evaluation metrics were added. Since the lane-level traffic state estimation task is formulated as a three-class classification problem, including Free, Saturated, and Congestion states, relying only on overall accuracy may be insufficient. In particular, when the sample distribution is imbalanced, the overall accuracy may be dominated by the majority class and may not fully reflect the model’s ability to identify more difficult traffic states.

**Figure 9:**
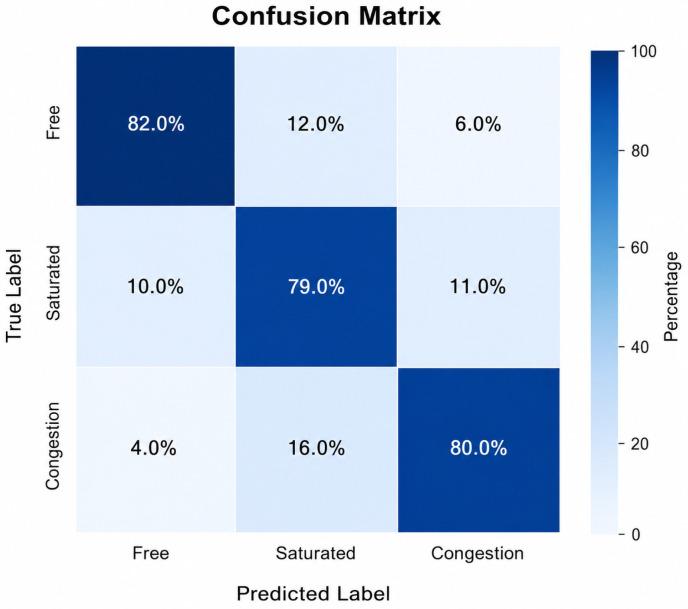
Normalized confusion matrix of the proposed GOT model.

Figure 9 presents the normalized confusion matrix of the proposed GOT model on the test set, where each row represents the distribution of predicted labels for samples belonging to a given true traffic state. The results indicate that the GOT model can correctly identify most samples in the three traffic states. Specifically, 82.0% of Free samples, 79.0% of Saturated samples, and 80.0% of Congestion samples are correctly classified. The Free state achieves the highest recognition performance, mainly because its traffic characteristics are relatively stable and distinguishable. The Congestion state is also recognized with satisfactory performance, suggesting that the model can capture low-velocity and high-density traffic patterns from microscopic trajectory features. The Saturated state shows relatively lower classification performance, mainly because it represents a transitional state between Free and Congestion. Therefore, some Saturated samples are misclassified as Free or Congestion when their microscopic motion characteristics are close to adjacent traffic states.

Table 7 further reports the precision, recall, and F1-score for each traffic state. The results show that the GOT model achieves relatively balanced performance across the three traffic states. Specifically, the F1-scores of Free, Saturated, and Congestion states are 0.84, 0.78, and 0.79, respectively. Although the Saturated state has a slightly lower F1-score, the model still maintains acceptable classification performance for this transitional state. These results further demonstrate that the proposed GOT model does not merely improve the overall accuracy but also maintains reasonable recognition capability for different traffic states.

Overall, the normalized confusion matrix and class-wise evaluation results provide a more comprehensive assessment of the proposed model. The results confirm that the GOT model performs reasonably well not only in terms of overall accuracy and validation loss but also in recognizing individual traffic states, including the more difficult Saturated and Congestion states.

## 5. Conclusions

This paper investigates lane-level traffic flow state estimation by explicitly linking microscopic vehicle trajectory data with macroscopic traffic flow states. Vehicle trajectory data describe individual vehicle-level motion characteristics, including position, velocity, acceleration, and temporal evolution, while traffic flow states reflect aggregated lane-level conditions such as traffic volume, average travel velocity, and headway. By extracting traffic flow indicators from vehicle trajectory records and analyzing vehicle motion patterns under different traffic states, this study demonstrates that microscopic vehicle trajectories contain informative cues for identifying macroscopic traffic flow conditions. Therefore, the study establishes a micro-to-macro connection between individual vehicle trajectory behavior and lane-level traffic flow state estimation.

To accommodate the requirements of trajectory-based traffic state estimation, we introduced a series of optimizations to the Transformer model, taking into account the nonlinear and locally fluctuating characteristics of microscopic vehicle trajectory data. First, the embedding layer was used to improve the representation of trajectory features. Second, the feed-forward network was extended to enhance nonlinear feature transformation. Third, a MaxPooling layer was added to preserve salient local trajectory responses and reduce feature redundancy. Finally, randomized search cross-validation was adopted for hyperparameter optimization, further improving model performance and generalization ability.

Through experiments on the proposed GOT model and benchmark models, including LSTM, RNN, and the basic Transformer, this paper validates the connection between microscopic vehicle trajectory features and lane-level traffic flow state categories. The experimental results show that the GOT model performs well in estimating Free, Saturated, and Congestion states from sparse microscopic trajectory observations. Compared with the benchmark models, the GOT model demonstrates a stronger ability to learn the nonlinear micro-to-macro mapping between individual vehicle motion patterns and aggregate traffic flow states. In addition to overall accuracy and validation loss, the normalized confusion matrix and class-wise precision, recall, and F1-score further confirm that the proposed GOT model achieves relatively balanced classification performance across different traffic states.

The main contribution of this paper lies in establishing a data-driven trajectory-based framework for lane-level traffic flow state estimation. By using microscopic vehicle trajectory data as model inputs and traffic flow states as estimation targets, this study provides a bridge between individual vehicle behavior analysis and macroscopic traffic flow monitoring. This not only enriches existing traffic management strategies but also provides a foundation for future research on the linkage between microscopic vehicle motion and macroscopic traffic flow evolution.

Nevertheless, the model performance in this study was evaluated on a single dataset, which may not fully represent diverse real-world traffic scenarios. Future research will further examine how the quality and availability of vehicle trajectory data influence lane-level traffic flow state estimation. In particular, the effects of vehicle density, road conditions, raw video quality, drone camera resolution, detection errors, trajectory sparsity, and adverse weather conditions will be considered. Moreover, the performance of other model structures, such as attention-based models and convolutional neural networks, will be evaluated on multiple datasets. These efforts will provide further support for more efficient and accurate trajectory-based traffic flow monitoring and management applications.

Nevertheless, the model performance in this study was evaluated on a single dataset, which may not fully represent diverse real-world traffic scenarios. Future research will further examine how the quality and availability of vehicle trajectory data influence lane-level traffic flow state estimation. In particular, the practical application of lane-level sparse trajectory estimation in real-world connected-vehicle environments will be further investigated. Under incomplete sensing conditions, such as low connected-vehicle penetration rates, partial trajectory availability, positioning errors, and communication delays, the robustness and reliability of the proposed framework need to be further evaluated. In addition, the effects of vehicle density, road conditions, raw video quality, drone camera resolution, detection errors, trajectory sparsity, and adverse weather conditions will be considered. Moreover, the performance of other model structures, such as attention-based models and convolutional neural networks, will be evaluated on multiple datasets. These efforts will provide further support for more efficient and accurate trajectory-based traffic flow monitoring and management applications.

## Figures and Tables

**Table 1:** Descriptive Statistics of Traffic Flow on the Examined Section.

Section Number	Traffic Volume (Veh/5 min)	Average Travel Velocity (km/h)	Headway (m)
Freeway 11 (17:20)	534	35.31	9.08
Freeway 12 (17:20)	405	81.45	66.30
Freeway 21 (17:25)	536	39.53	10.64
Freeway 22 (17:25)	441	72.61	64.98
Freeway 31 (17:30)	489	39.49	32.71
Freeway 32 (17:30)	440	75.12	50.66
Freeway 41 (17:48)	519	29.27	11.74
Freeway 42 (17:48)	437	71.86	61.20
Freeway 51 (17:53)	560	33.86	11.27
Freeway 52 (17:53)	419	79.05	59.39
Freeway 61 (17:58)	504	23.54	7.36
Freeway 62 (17:58)	420	78.54	52.32
Freeway 71 (18:03)	472	38.24	26.14
Freeway 72 (18:03)	419	85.10	78.08

**Table 2:** Traffic Statistics Status.

Traffic Status Categories	Traffic Volume (veh/5 min)	Average Travel Velocity (km/h)	Headway (m)	Included Data Sets
Free	410	77.7	61.8	Freeway 52, Freeway 62, Freeway 72
Saturated	475	33.8	22.1	Freeway 22, Freeway 32, Freeway 42
Congestion	525	34.5	10.7	Freeway 21, Freeway 31, Freeway 41

**Table 3:** Basic Statistical Analysis of Vehicle Velocity and Acceleration.

Base Statistic.	Velocity			Acceleration		
	Free	Saturated	Congestion	Free	Saturated	Congestion
Mean	51.26	46.60	22.68	−0.27	−0.18	0.17
Median	50.92	45.56	21.48	−0.20	−0.10	0.14
Skewness	0.16	0.36	0.49	−3.66	−2.69	0.91
Kurtosis	2.60	2.61	2.37	20.53	27.11	11.18
Jarque–Bera estimate	616.29	716.37	903.36	9209.31	6517.66	4500.21
Maximum value	74.76	69.04	38.79	1.38	3.94	2.95
Minimum value	31.24	27.37	10.89	−4.35	−4.35	−2.07
10th Percentile	40.75	36.11	17.30	−0.65	−0.54	−0.15
25th Percentile	45.07	40.66	18.43	−0.39	−0.27	0.01
75th Percentile	56.97	52.07	26.64	−0.03	0.03	0.34
90th Percentile	61.96	59.02	30.34	0.12	0.16	0.50
Quartile range	11.90	11.41	8.21	0.36	0.30	0.32
Standard Deviation	8.37	8.51	5.14	0.53	0.50	0.32
Mean Absolute Deviation	6.84	6.91	4.37	0.30	0.26	0.22
Coefficient of Variation	0.16	0.18	0.23	1.96	2.83	1.90

Note: The statistics of velocity, including mean, median, maximum, minimum, percentiles, quartile range, standard deviation, and mean absolute deviation, are reported in km/h. The corresponding statistics of acceleration are reported in m/s^2^. Skewness, kurtosis, Jarque–Bera estimate, and coefficient of variation are dimensionless.

**Table 4:** Detailed experimental parameter settings of benchmark models and the proposed GOT model.

Model	Main Structure	Layers/Blocks	Hidden Units/FFN Units	Attention Heads	Dropout	Optimizer	Learning Rate	Epochs	Batch Size
LSTM	LSTM + FC layers	1 LSTM layer + 2 FC layers	LSTM: 100; FC: 64; Output: 3	N/A	N/A	Adam	0.001	20	32
RNN	Simple RNN + FC layer	2 RNN layers + 1 FC layer	RNN: 128 each; Output: 3	N/A	0.5	RMSProp	0.001	10	64
Transformer	Transformer encoder + global average pooling + FC	2 Transformer blocks	FFN: 64; Output: 3	4	0.1	RMSprop	0.001	10	16
GOT	Transformer encoder + extended FFN + MaxPooling + FC	2 Transformer blocks + 3-layer FFN	FFN: 128–64–32; Output: 3	4	0.1	SGD	0.001	10	16

**Table 5:** Ablation study of the proposed GOT model.

Model	Accuracy (%)	Validation Loss
Basic Transformer	76.85	0.462
Transformer + 3rd FFN	78.12	0.438
Transformer + MaxPooling	78.64	0.421
GOT	80.23	0.387

**Table 6:** Sensitivity analysis of different pooling strategies.

Pooling Strategy	Accuracy (%)	Validation Loss
No Pooling	78.12	0.438
Average Pooling	78.57	0.426
MaxPooling	80.23	0.387
Attention-based Pooling	79.84	0.395

**Table 7:** Class-wise evaluation metrics of the proposed GOT model.

Traffic State	Precision	Recall	F1-Score
Free	0.86	0.82	0.84
Saturated	0.77	0.79	0.78
Congestion	0.78	0.80	0.79
Macro Average	0.80	0.80	0.80
Weighted Average	0.81	0.80	0.80

## Data Availability

The authors do not have permission to share.
